# Diketoacetonylphenalenone, Derived from Hawaiian Volcanic Soil-Associated Fungus *Penicillium herquei* FT729, Regulates T Cell Activation via Nuclear Factor-κB and Mitogen-Activated Protein Kinase Pathway

**DOI:** 10.3390/molecules25225374

**Published:** 2020-11-17

**Authors:** Hyun-Su Lee, Jae Sik Yu, Ki Hyun Kim, Gil-Saeng Jeong

**Affiliations:** 1 College of Pharmacy, Keimyung University, Daegu 42601, Korea; hyunsu.lee@kmu.ac.kr; 2 School of Pharmacy, Sungkyunkwan University, Suwon 16419, Korea; jsyu@bu.edu

**Keywords:** T cells, *Penicillium herquei* FT729, immunosuppressive

## Abstract

In immunological responses, controlling excessive T cell activity is critical for immunological homeostasis maintenance. Diketoacetonylphenalenone, derived from Hawaiian volcanic soil-associated fungus *Penicillium herquei* FT729, possesses moderate anti-inflammatory activity in RAW 264.7 cells but its immunosuppressive effect on T cell activation is unknown. In the present study, diketoacetonylphenalenone (up to 40 μM) did not show cytotoxicity in T cells. Western blot analysis showed treatment with diketoacetonylphenalenone did not alter the expression of anti-apoptotic proteins. Pretreatment with diketoacetonylphenalenone suppressed the interleukin-2 production in activated T cells induced by T cell receptor-mediated stimulation and PMA/A23187. The CFSE-proliferation assay revealed the inhibitory effect of diketoacetonylphenalenone on the proliferation of T cells. The expression of surface molecules on activated T cells was also reduced. We discovered the suppression of the TAK1-IKKα-NF-κB pathway by pretreatment with diketoacetonylphenalenone abrogated mitogen-activated protein kinase (MAPK) signaling in activated T cells. These results suggest that diketoacetonylphenalenone effectively downregulates T cell activity via the MAPK pathway and provides insight into the therapeutic potential of immunosuppressive reagents.

## 1. Introduction

The activity of T cells plays a central role in inflammatory conditions in which primed T cells generate several effector cytokines during proliferation or differentiation [1]. Initial contact between naïve T cells and antigen presenting cells (APCs) provokes priming signals that induce antigen specificity through T cell receptor (TCR)-major histocompatibility complex (MHC) II ligation [2]. A TCR-mediated signal is rapidly transduced to the nucleus and results in changes in gene expression. Interleukin (IL)-2 has been studied as an early marker of T cell activation as IL-2 binds to the IL-2 receptor (CD25) to provide potent activation signals (autocrine and paracrine signaling), which leads to T cell proliferation after priming [3]. CD69 is expressed on the surface of T cells in the early stages after TCR stimulation and plays a critical role in the regulation of T cell differentiation into regulatory T cells and secretion of IL-17, interferon-γ, and IL-22 [4]. The early signaling pathway of T cell activation promotes T cell proliferation and differentiation into effector T cells that produce effector cytokines. As the initial T cell activation is a key event in the inflammatory response, the importance of immunosuppressive reagents to maintain immunological homeostasis is becoming increasingly appreciated.

Transforming growth factor-β-activated kinase 1 (TAK1) has been studied as a central regulator of cellular responses, including activation, inflammation, and death [5,6]. Several studies have reported that TAK1 can be activated by tumor necrosis factor-α and IL-1β through their receptors, lipopolysaccharides via Toll-like receptor 4, and TCR signaling [6,7,8,9]. It has also been elucidated as an upstream modulator of nuclear factor (NF-κB), which is the most important transcription factor for T cell activation [10]. In the TCR-mediated signaling pathway, the phosphorylation of TAK1 leads to the formation of the IκB kinase (IKK) complex, which consists of two subunits (IKKα and IKKβ) and one inhibitory unit (the NF-κB essential modulator) and activation of its catalytic action to phosphorylate the inhibitor of NF-κB—IκBα. Phosphorylated IκBα, which is eventually degraded by proteasomes, promotes the translocation of the p65 subunit of NF-κB and plays a role as a central transcription factor in response to TCR stimulation. TAK1 activation has also been shown to be involved in the activation of the MAPKs, including extracellular signal-regulated kinase (ERK), p38, and c-Jun N-terminal kinase (JNK) [5]. Based on these reports, TAK1 is widely considered as a molecular target for inflammatory diseases and therapeutic candidate for the development of immunosuppressive reagents [11].

*Penicillium herquei* is a fungus, isolated from soil and marine organisms, that possesses several bioactive secondary metabolites, such as inhibitors of the aggregation of blood platelets (herquline A and B) [12] and various phenalenone antibiotics (herqueichrysin, deoxyherqueinone, and atrovenetin) [13]. Two new steroids and a urea derivative have also been isolated from *P. herquei* [14,15]. Some metabolites derived from *P. herquei* have shown significant antifungal and anti-influenza activities as well as cytotoxic activity against MDA-ME-231 and MV4-11 cells [16,17,18]. A recent report has demonstrated that the acetone adduct of a triketone (we forward named it as diketoacetonylphenalenone) successfully isolated from marine-derived *Penicillium sp.* shows anti-inflammatory activity in RAW 264.7 cells [19]; however, little is known about its immunosuppressive effect on T cell activation. In the present study, a soil-associated fungal strain *P. herquei* FT729 was isolated from a soil sample collected at Hualālai, an active volcano on the Island of Hawaii. As a part of ongoing projects to discover bioactive compounds from natural sources [20,21,22,23,24,25,26,27], chemical investigation of the Hawaiian volcanic soil-associated fungus *P. herquei* FT729 led to the isolation of a phenalenone derivative by LC/MS-guided chemical analysis. Herein, we described the isolation and structural characterization of the phenalenone derivative, and explored whether the compound has toxicity in T cells and modulatory functions in early T cell activation in vitro.

## 2. Results

### 2.1. Isolation and Identification of Diketoacetonylphenalenone from Penicillium Herquei FT729

**Figure 1:**
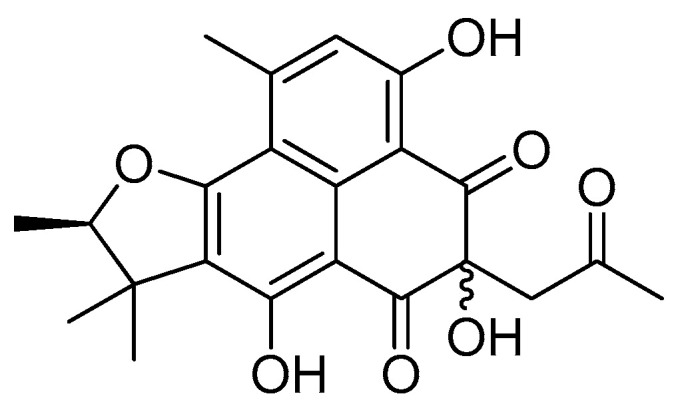
The chemical structure of diketoacetonylphenalenone from *P. herquei* FT729.

LC/MS-based chemical analysis of a MeOH extract of *P. herquei* FT729, using successive column chromatography over silica gel, RP-C_18_ silica, and Sephadex LH-20, along with preparative and semi-preparative HPLC resulted in the isolation and identification of a phenalenone derivative from the EtOAc soluble fraction. Its structural elucidation (Figure 1) was confirmed by comparing their optical rotation value and NMR spectra data (Supplementary Material) with those reported earlier [19,28], and LC/MS analysis. The isolated compound was previously reported as acetone adduct of a triketone [19,28], and we named it as diketoacetonylphenalenone.

### 2.2. Diketoacetonylphenalenone Is Not Cytotoxic to T Cells

**Figure 2:**
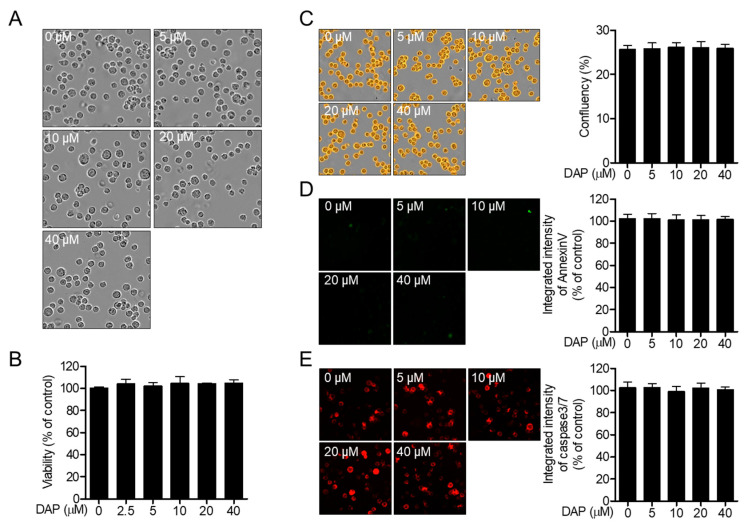
Diketoacetonylphenalenone (DAP) is not cytotoxic to T cells. (**A**) Differential interference contrast (DIC) microscopic images of Jurkat T cells treated with the indicated concentration of diketoacetonylphenalenone (0–40 μM) for 24 h. (**B**) Cell viability of Jurkat T cells treated with the indicated concentration of diketoacetonylphenalenone (0–40 μM) for 24 h using the MTT assay. (**C**–**E**) Jurkat T cells were treated with the indicated concentration of diketoacetonylphenalenone (0–40 μM) for 24 h and the confluency (**C**), intensity of Annexin V (**D**), and intensity of caspase 3/7 (**E**) were determined using the IncuCyte^®^ imaging system. In the treatment with diketoacetonylphenalenone, cells were cotreated with 1 × Annexin V staining reagent and caspase 3/7 staining reagent. Cell confluency and the integrated fluorescence intensity of Annexin V and caspase3/7 were automatically calculated in IncuCyte^®^ software and bar graphs show % of control. The mean value of three experiments ± SEM is presented. Abbreviations: MTT, 1-(4,5-dimethylthiazol-2-yl)-3,5-diphenylformazan.

We first examined whether diketoacetonylphenalenone affects the cytotoxicity of T cells. Treatment with diketoacetonylphenalenone caused no significant cellular morphological changes in Jurkat T cells after incubation for 24 h (Figure 2A). Cell viability was assessed using 1-(4,5-dimethylthiazol-2-yl)-3,5-diphenylformazan (MTT) after treatment with diketoacetonylphenalenone (Figure 2B). To evaluate whether treatment with diketoacetonylphenalenone activates the apoptotic pathway, the fluorescence intensities of Annexin V and caspase 3/7 were observed using the IncuCyte^®^ imaging system. The confluency of T cells was not altered after incubation with diketoacetonylphenalenone (Figure 2C). The fluorescence of Annexin V was not detected but the intensity of expressed caspase 3/7 did not change in the presence of diketoacetonylphenalenone (Figure 2D,E). These results suggest that treatment with diketoacetonylphenalenone is not cytotoxic to Jurkat T cells.

### 2.3. Treatment with Diketoacetonylphenalenone Does Not Change the Expression of Anti-Apoptotic Proteins

**Figure 3:**
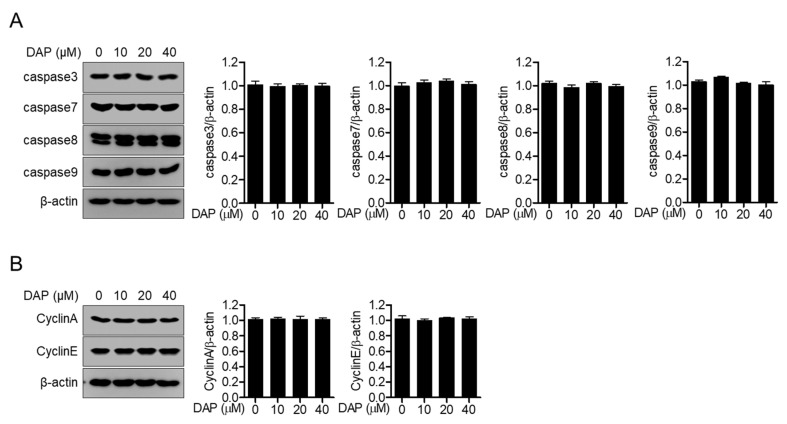
Treatment with diketoacetonylphenalenone (DAP) does not change the expression of anti-apoptotic proteins. (**A**) Expression of the indicated caspase proteins in Jurkat T cells treated with the indicated concentration of diketoacetonylphenalenone (0–40 μM) for 24 h by Western blot analysis. (**B**) Expression of the indicated cyclin proteins in Jurkat T cells treated with the indicated concentration of diketoacetonylphenalenone (0–40 μM) for 24 h by Western blot analysis. The detected bands were normalized to the expression of β-actin and the mean value of three experiments ± SEM is presented in the bar graph.

To confirm whether diketoacetonylphenalenone is not associated with the apoptotic pathway in Jurkat T cells, the expression of anti-apoptotic proteins, such as caspase 3, caspase 7, caspase 8, and caspase 9 was detected in Jurkat T cells incubated with diketoacetonylphenalenone. The expression of these proteins was observed in Jurkat T cells treated with up to 40 μM diketoacetonylphenalenone (Figure 3A). The expression of cyclins (cyclin A and cyclin E) was also measured to examine whether treatment with diketoacetonylphenalenone is involved in the cell cycle. Incubation with diketoacetonylphenalenone did not arrest the cell cycle in Jurkat T cells (Figure 3B). These data suggest that diketoacetonylphenalenone does not activate the apoptotic pathway nor induce cell cycle arrest in Jurkat T cells.

### 2.4. Pretreatment with Diketoacetonylphenalenone Inhibits IL-2 Production from Activated T Cells

**Figure 4:**
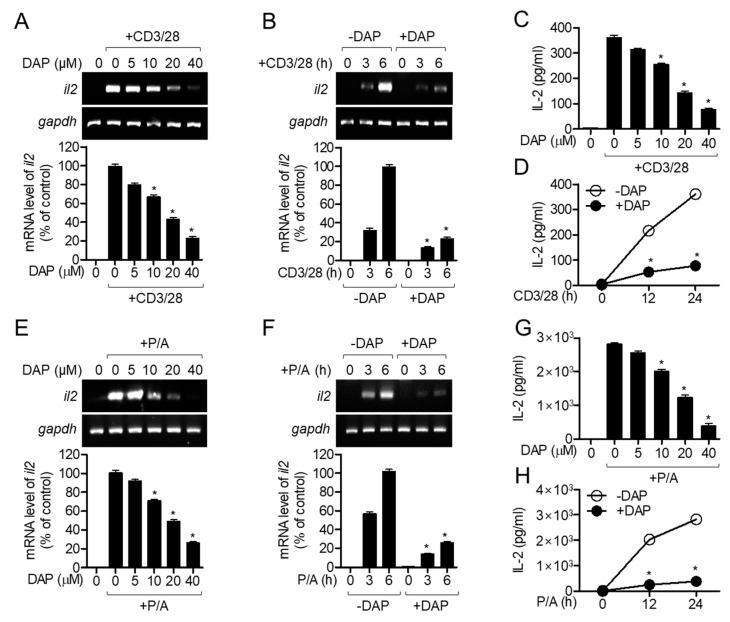
Pretreatment with diketoacetonylphenalenone (DAP) inhibits IL-2 production from activated T cells. (**A**,**B**) Jurkat T cells pretreated with the indicated concentration of diketoacetonylphenalenone (0–40 μM) (**A**) or 40 μM (**B**) were stimulated with anti-CD3 (20 μg/mL)/anti-CD28 (7 μg/mL) for 6 h (**A**) or the indicated time (0–6 h) (**B**). After stimulation, the mRNA level of *il2* was measured by conventional and quantitative PCR from harvested cells. (**C**,**D**) Jurkat T cells pretreated with the indicated concentration of diketoacetonylphenalenone (0–40 μM) (**C**) or 40 μM (**D**) were stimulated with anti-CD3 (20 μg/mL)/anti-CD28 (7 μg/mL) for 24 h (**C**) or the indicated time (0–24 h) (**D**). Amount of IL-2 produced was measured by ELISA from supernatants. (**E**,**F**) Jurkat T cells pretreated with the indicated concentration of diketoacetonylphenalenone (0–40 μM) (**A**) or 40 μM (**B**) were stimulated with PMA (100 nM)/A23187 (1 μM) for 6 h (**E**) or the indicated time (0–6 h) (**F**). mRNA level of *il2* was measured by conventional and quantitative PCR from harvested cells. (**G**,**H**) Jurkat T cells pretreated with the indicated concentration of diketoacetonylphenalenone (0–40 μM) (**G**) or 40 μM (**H**) were stimulated with PMA (100 nM)/A23187 (1 μM) for 24 h (**G**) or the indicated time (0–24 h) (**H**). After stimulation, supernatants were harvested and the amount of IL-2 produced was measured by ELISA. The mean value of three experiments ± SEM is presented. * *p* < 0.05 compared with mock-treated cells. Abbreviations: ELISA, enzyme-linked immunosorbent assay; IL, interleukin; PMA, phorbol 12-myristate 13-acetate.

IL-2 is a marker of the early activation of T cells as it is required for T cell proliferation and differentiation into effector T cells after priming from APCs. To evaluate whether pretreatment with diketoacetonylphenalenone regulates TCR-mediated T cell activation, the mRNA levels of *il2* were measured by conventional and quantitative PCR. The mRNA levels of *il2* in Jurkat T cells pretreated with diketoacetonylphenalenone decreased in a dose-dependent manner (Figure 4A). Time-dependent experiments also revealed that pretreatment with diketoacetonylphenalenone suppressed the mRNA levels of *il2* in Jurkat T cells (Figure 4B). To show the inhibitory effect of diketoacetonylphenalenone on the induction of *il2* gene expression, we measured the amount of IL-2 produced by activated T cells using anti-CD3/CD28 antibodies in the pretreatment with diketoacetonylphenalenone. The results of the enzyme-linked immunosorbent assay (ELISA) showed that pretreatment with diketoacetonylphenalenone suppressed IL-2 production in activated Jurkat T cells in a dose- and time-dependent manner (Figure 4C,D). As treatment with phorbol 12-myristate 13-acetate (PMA)/A23187 is a widely used method to stimulate IL-2 production by T cells, we investigated whether pretreated PMA/A23187-stimulated Jurkat T cells generate less IL-2. Pretreatment with diketoacetonylphenalenone significantly abrogated the induction of *il2* expression in PMA/A23187-stimulated Jurkat T cells (Figure 4E,F). The ELISA also showed that pretreatment with diketoacetonylphenalenone downregulated IL-2 production in Jurkat T cells stimulated with PMA/A23187. These data suggest that pretreatment with diketoacetonylphenalenone effectively controls T cell activation in terms of IL-2 production at the mRNA and protein levels.

### 2.5. Pretreatment with Diketoacetonylphenalenone Inhibits Activated T Cell Proliferation

**Figure 5:**
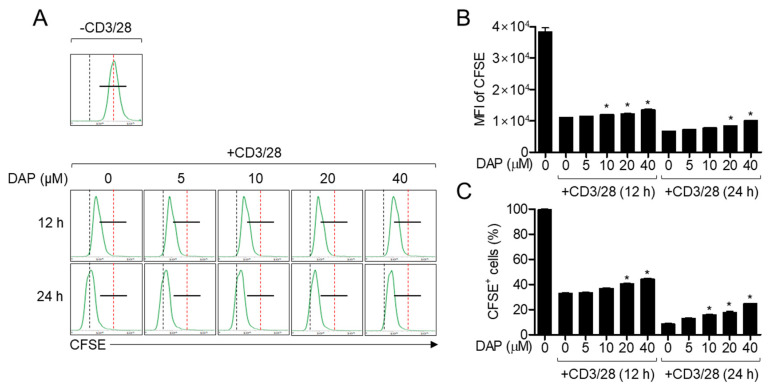
Pretreatment with diketoacetonylphenalenone (DAP) inhibits activated T cell proliferation. (**A**) Jurkat T cells pre-stained with 1 μM CFSE for 30 min were pretreated with the indicated concentration of diketoacetonylphenalenone for 1 h. Cells were then stimulated with anti-CD3 (20 μg/mL)/CD28 (7 μg/mL) antibodies for 12 h and 24 h and harvested for flow cytometry analysis. Black line indicates the segmentation of CFSE-positive cells from positive control (-CD3/CD28). Black dot line means the peak of stimulated cells for 24 h (lowest value) and red dot line represents the peak of resting cells (highest value). (**B**) Mean fluorescence intensity of CFSE-stained cells from Figure 5A. (**C**) Percentage of CFSE-positive cells from Figure 5A. The mean value of three experiments ± SEM is presented. * *p* < 0.05 compared with mock-treated cells. Abbreviations: CFSE, carboxyfluorescein succinimidyl ester.

Because the expression of IL-2 is pivotal for T cell proliferation, we elucidated whether pretreatment with diketoacetonylphenalenone has a regulatory effect on TCR-mediated T cell proliferation. The number of Jurkat T cells stained with carboxyfluorescein succinimidyl ester (CFSE) was gradually reduced owing to proliferation in a time-dependent manner (Figure 5A). However, pretreatment with diketoacetonylphenalenone blocked the reduction of CFSE-stained Jurkat T cells in a dose-dependent manner. Furthermore, pretreatment with diketoacetonylphenalenone reduced the decrement of mean fluorescence intensity (MFI) of CFSE and abrogation of CFSE-positive cell population by TCR-mediated stimulation (Figure 5B,C). These results suggest that T cell proliferation is affected by pretreatment with diketoacetonylphenalenone in a dose-dependent manner.

### 2.6. Pretreatment with Diketoacetonylphenalenone Suppresses the Expression of Surface Molecules on Activated T Cells

**Figure 6:**
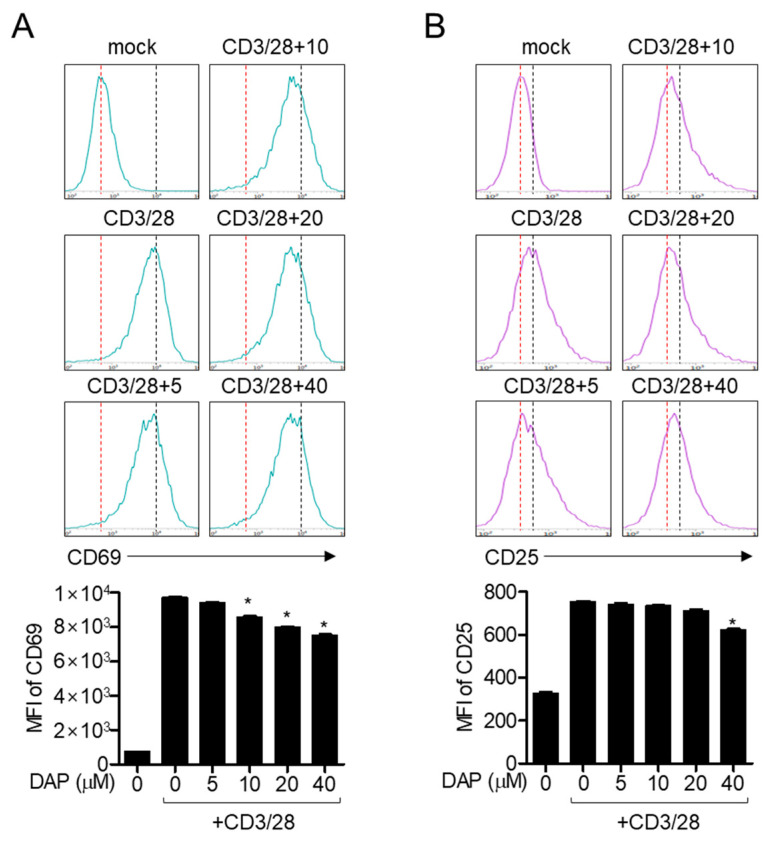
Pretreatment with diketoacetonylphenalenone (DAP) suppresses the expression of surface molecules on activated T cells. (**A**,**B**) Jurkat T cells pretreated with the indicated concentration of diketoacetonylphenalenone for 1 h were stimulated with anti-CD3 (20 μg/mL)/CD28 (7 μg/mL) antibodies for 16 h (**A**) or 24 h (**B**). Harvested cells were stained with anti-CD69 antibodies to detect CD69 expression or with anti-CD25 antibodies to detect CD25 expression by flow cytometry. Red dot line represents the peak of resting cells (negative control) and black dot line indicates the peak of activated cells (positive control). The mean value of three experiments ± SEM is presented. * *p* < 0.05 compared with mock-treated cells.

As the level of CD69 has been reported to rapidly increase after TCR-mediated stimulation, it is widely used as a marker of the early activation of T cells. To determine whether the expression of CD69 on the surface of activated Jurkat T cells after TCR-mediated stimulation is suppressed by pretreatment with diketoacetonylphenalenone, CD69 was measured by flow cytometry. The increase in CD69 expression induced by anti-CD3/CD28 antibodies was significantly inhibited by pretreatment with diketoacetonylphenalenone in a dose-dependent manner (Figure 6A). The IL-2 dependent autocrine pathway plays a pivotal role in T cell proliferation and growth. We further measured the expression of CD25, the IL-2 receptor, to examine whether pretreatment with diketoacetonylphenalenone affects the expression of CD25. CD25 expression on activated T cells after TCR-mediated stimulation was slightly downregulated by pretreatment with diketoacetonylphenalenone (Figure 6B). These flow cytometry results suggest that diketoacetonylphenalenone partially regulates CD69 and CD25 expression in activated T cells.

### 2.7. Pretreatment with Diketoacetonylphenalenone Inhibits the MAPK Signaling Pathway via the Regulation of the TAK1-IKKα-NF-κB Pathway

**Figure 7:**
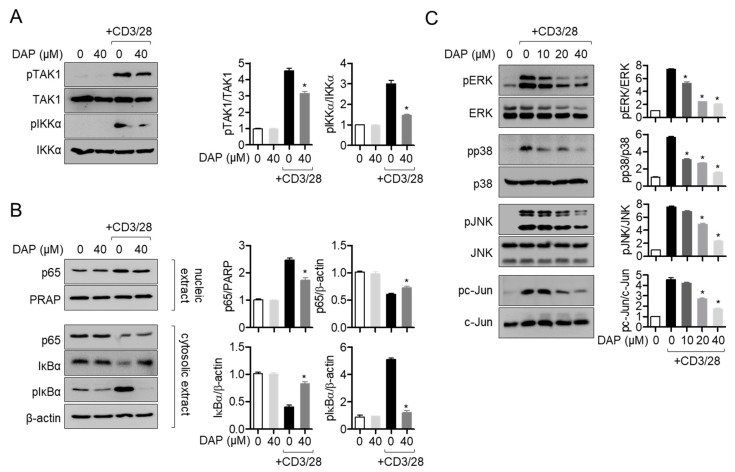
Pretreatment with diketoacetonylphenalenone (DAP) inhibits the MAPK signaling pathway via the regulation of the TAK1-IKKα-NF-κB pathway. (**A**) Jurkat T cells pretreated with 40 μM diketoacetonylphenalenone for 1 h were stimulated with anti-CD3 (20 μg/mL)/CD28 (7 μg/mL) for 15 min and harvested for lysis. The indicated proteins were detected by Western blot analysis. The levels of phosphorylated TAK1 and IKKα were quantified by the expression of TAK1 and IKKα. (**B**) Jurkat T cells pretreated with 40 μM diketoacetonylphenalenone for 1 h were stimulated with anti-CD3 (20 μg/mL)/CD28 (7 μg/mL) for 1 h and harvested for lysis. Nuclear extracts were separated from whole lysates using the NE-PER kit and the indicated proteins in nuclear and cytosolic extracts were detected by Western blot analysis. p65 translocation was measured by comparing the detected p65 in nuclear and cytosolic extracts. The levels of phosphorylated IκBα and IKKα were also detected. Detected bands were normalized to the expression of PARP for nuclear extracts and β-actin for cytosolic extracts. (**C**) Jurkat T cells pretreated with the indicated concentration of diketoacetonylphenalenone for 1 h were stimulated with anti-CD3 (20 μg/mL)/CD28 (7 μg/mL) for 30 min and harvested for lysis. The indicated proteins were detected by Western blot analysis. The levels of phosphorylated ERK, p38, JNK, and c-Jun were quantified by the expression of TAK1 and IKKα. The mean value of three experiments ± SEM is presented. * *p* < 0.05 compared with mock-treated cells. Abbreviations: ERK, extracellular signal-regulated kinase; IKK, IκB kinase; JNK, c-Jun N-terminal kinase; TAK, transforming growth factor-β-activated kinase.

TAK1 has been studied as a modulator of IKKα, which plays a critical role in the activation of the NF-κB pathway via the TCR signaling pathway. To evaluate the underlying inhibitory mechanism of diketoacetonylphenalenone on T cell activation, we determined the phosphorylation of TAK1 and IKKα after TCR-mediated stimulation. Western blot analysis showed that pretreatment with diketoacetonylphenalenone regulated the phosphorylation of TAK1 in activated T cells (Figure 7A). The phosphorylation of IKKα was reduced in activated Jurkat T cells pretreated with diketoacetonylphenalenone. We further investigated whether the translocation of p65, a subunit of NF-κB, into the nucleus is affected by the reduction of TAK1-IKKα phosphorylation by Western blotting. The translocation of p65 into the nucleus was regulated by pretreatment with diketoacetonylphenalenone after TCR-mediated stimulation (Figure 7B). Consequently, the degradation and phosphorylation of IκBα were also abrogated by pretreatment with diketoacetonylphenalenone. As the MAPK pathway, the most important signaling pathway for T cell activation in terms of IL-2 production, is downstream of the NF-κB pathway, we determined the phosphorylation levels of ERK, p38, JNK, and c-Jun from activated T cells pretreated with diketoacetonylphenalenone. Western blot analysis revealed that pretreatment with diketoacetonylphenalenone suppressed the phosphorylation of ERK, p38, JNK, and c-Jun in activated T cells induced by TCR-mediated stimulation (Figure 7C). These results suggest that diketoacetonylphenalenone controls the MAPK pathway in activated T cells through the inhibition of the TAK1-IKKα-NF-κB pathway.

## 3. Discussion

In the current study, we examined the modulatory effect of diketoacetonylphenalenone isolated from *P. herquei* FT729 on T cell activation. Biochemical analysis revealed that treatment with diketoacetonylphenalenone up to 40 μM did not lead to the apoptotic pathway in T cells, arrest the T cell cell cycle, or result in cytotoxicity. Pretreatment with diketoacetonylphenalenone significantly reduced the production of IL-2, expression of surface molecules including CD69 and CD25, proliferation of activated T cells after TCR-mediated stimulation and abrogated the NF-κB and MAPK pathways. These data suggest the potential of diketoacetonylphenalenone as an immunosuppressive reagent to balance T cell homeostasis.

Among several established methods to stimulate T cells in vitro, TCR-mediated stimulation by antibodies and stimulators which bypass TCR ligation with MHCII were used in the present study. Using anti-CD3/CD28 antibodies for T cell stimulation is a classical method in which TCRs are tightly accumulated by immobilized anti-CD3 antibodies and co-stimulatory signals are produced by anti-CD28 antibodies. This method physically mimics the ligation between TCR and MHC molecules in vivo. Another method to stimulate T cells used in this study is treatment with PMA, a protein kinase C (PKC) activator, and A23187, a calcium ionophore. Because PMA directly activates PKC, which is a downstream modulator of TCRξ phosphorylation, and A23187 induces calcium influx into the cytosol, this method systemically mimics the cellular response after TCR-mediated stimulation. We demonstrated the inhibitory effect of diketoacetonylphenalenone on T cell activation by anti-CD3/CD28 and PMA/A23187 treatment.

TCR signaling is involved in T cell proliferation via IL-2 signaling. An ex vivo study demonstrated that the loss-of-function of IL-2 during T cell activation results in a severe lack of T cell proliferation [29]. Furthermore, in addition to the amount of IL-2, the expression of the IL-2 receptor is important for T cell proliferation. A previous report revealed that the blockade of the IL-2 receptor by neutralizing antibodies leads to abrogated T cell proliferation. [30]. The current study showed not only the regulatory effect of diketoacetonylphenalenone on IL-2 production (Figure 4), but also the downregulation of the expression of CD25 in a dose-dependent manner (Figure 6). Additionally, the CFSE proliferation assay revealed that pretreatment with diketoacetonylphenalenone controls T cell proliferation in response to TCR-mediated stimulation. However, it would be beneficial for the effect of diketoacetonylphenalenone on T cell differentiation into effector T cells to be examined.

The development of therapeutics for T cell-mediated diseases, including autoimmune diseases and graft-versus-host disease (GVHD), is needed. Cyclosporin A, a representative immunosuppressant for GVHD, rheumatoid arthritis, Crohn’s disease, and systemic lupus erythematosus, was first isolated from *Cylindrocarpon lucidum* [31,32]. Tremendous effort is still required to uncover potent naturally-derived immunosuppressive reagents, such as tacrolimus, that are effective with low cytotoxicity [33]. Here, we evaluated the potential of diketoacetonylphenalenone isolated from *P. herquei* as an immunosuppressant and observed that it effectively regulates T cell activity without cytotoxicity in vitro. Further studies should include animal experiments to validate the preventive effect diketoacetonylphenalenone on T cell-mediated diseases such as atopic dermatitis and inflammatory bowel disease.

## 4. Materials and Methods

### 4.1. Cells

Jurkat T cells (KCLB number: 40152), purchased from Korean Cell Line Bank (Seoul, Korea), were cultured in RPMA medium (Welgene, Gyeongsan, Korea) supplemented with streptomycin (100 μg/mL), penicillin G (100 unit/mL), 10% fetal bovine serum (FBS), and L-glutamine (2 mM) at 37 °C in a humidified incubator containing 5% CO_2_. The cells were passaged five to eight times for the experiments.

### 4.2. General Experiment Procedures

Optical rotations were measured using a Jasco P-2000 polarimeter (Jasco, Easton, MD, USA). Infrared (IR) spectra were acquired on a JASCO Fourier Transform Infrared (FT/IR)-4600 spectrometer (JASCO, Easton, MD, USA). Ultraviolet (UV) spectra were acquired on an Agilent 8453 UV-visible spectrophotometer (Agilent Technologies, Santa Clara, CA, USA). Nuclear magnetic resonance (NMR) spectra were recorded using a Bruker AVANCE III HD 850 NMR spectrometer with a 5 mm TCI CryoProbe operating at 850 MHz (^1^H) and 212.5 MHz (^13^C), for ^1^H and ^13^C NMR analyses, respectively. Chemical shifts are reported in ppm using the residual solvent peaks as reference: CD_3_OD, δ = 3.34 ppm (^1^H NMR) and δ = 49.1 ppm (^13^C NMR). Preparative high-performance liquid chromatography (HPLC) was performed using a Waters 1525 Binary HPLC pump with a Waters 996 Photodiode Array Detector (Waters Corporation, Milford, MA, USA) and an Agilent Eclipse C_18_ column (250 × 21.2 mm, 5 μm; flow rate: 5 mL/min; Agilent Technologies). Semi-preparative HPLC was performed using a Shimadzu Prominence HPLC System with SPD-20A/20AV Series Prominence HPLC UV-Vis detectors (Shimadzu, Tokyo, Japan) and a Phenomenex Luna Phenyl-Hexyl column (250 × 10 mm, 10 μm; flow rate: 2 mL/min; Phenomenex, Torrance, CA, USA). LC/MS analysis was performed on an Agilent 1200 Series HPLC system equipped with a diode array detector and 6130 Series ESI mass spectrometer using an analytical Kinetex C_18_ 100 Å column (100 × 2.1 mm, 5 μm; flow rate: 0.3 mL/min; Phenomenex). Silica gel 60 (230–400 mesh; Merck, Darmstadt, Germany) and RP-C_18_ silica gel (Merck, 230–400 mesh) were used for column chromatography. The packing material for molecular sieve column chromatography was Sephadex LH-20 (Pharmacia, Uppsala, Sweden). Thin-layer chromatography (TLC) was performed with precoated silica gel F_254_ plates and RP-C_18_ F_254s_ plates (Merck); the spots were detected under UV light or by heating after spraying with anisaldehyde-sulfuric acid.

### 4.3. Fungal Material and Cultivation

The fungal strain was isolated on PDA medium from a soil sample collected on Big Island, Hawaii in 2014. The strain FT729 was identified to be *Penicillium herquei* on the basis of DNA sequence analysis of the nuclear ribosomal internal transcribed spacer, which has been deposited in GenBank with the accession no. MN817943. A voucher specimen was deposited at Daniel K. Inouye College of Pharmacy, University of Hawaii at Hilo, USA (accession no. FT729). The fungus was cultured under static conditions at room temperature for 30 days in five conical flasks (1 L) containing 300 mL/flask liquid medium. The medium was composed of mannitol (20 g), glucose (10 g), monosodium glutamate (5 g), KH_2_PO_4_ (0.5 g), MgSO_4_·7H_2_O (0.3 g), yeast extract (3 g), corn steep liquor (2 mL) in 1 L distilled water at pH 6.5 prior to sterilization.

### 4.4. Isolation and Purification of Diketoacetonylphenalenone from P. Herquei FT729

Both mycelia and broth liquid solutions of *P. herquei* FT729 were first examined through LC/MS analysis and showed no significant difference. Thus, both were combined and concentrated with 80% MeOH/H_2_O under reduced pressure using a rotavapor to obtain 12.5 g of crude extract. The crude extract was suspended in distilled water and subsequently solvent-partitioned by liquid-liquid extraction with ethyl acetate (EtOAc) and *n*-butanol (BuOH). Two layers of increasing polarity, EtOAc-soluble (3.8 g) and BuOH-soluble (1.0 g) fractions, were obtained. Both fractions were screened through LC/MS analysis using our in-house UV library, and the EtOAc-soluble fraction was considered a significant target for isolation. The EtOAc-soluble fraction (3.8 g) was fractionated using silica gel column chromatography [eluted with CH_2_Cl_2_/MeOH (100:1→1:1→100% MeOH) gradient system] to obtain seven fractions (A1–A10). Fraction A3 (935.1 mg) was subjected to Sephadex LH-20 [eluted with CH_2_Cl_2_/MeOH (1:1) isocratic system] to obtain six subfractions (A31–A36). Subfraction A33 (178.3 mg) was fractionated with preparative reversed-phase HPLC with MeOH/H_2_O (3/5→100% MeOH) gradient system to produce four subfractions A331–334. Subfraction A333 (98.1 mg) was purified using semi-preparative HPLC (58% MeCN) to give diketoacetonylphenalenone (*t_R_* 32.5 min, 55.5 mg).

### 4.5. Reagents and Antibodies

MTT powder, PMA, and A23187 were purchased from Sigma Chemical Co. (St. Louis, MO, USA). Annexin V and caspase 3/7 staining reagents for the IncuCyte^®^ cell imaging system were obtained from Essen Bio (Ann Arbor, MI, USA). Human IL-2 ELISA kits were purchased from R&D (Minneapolis, MN, USA). CFSE dye, enhanced chemiluminescence (ECL) Western blotting detection reagents, and the NE-PER Nuclear and Cytoplasmic Extraction Reagents Kit were obtained from Thermo Fisher Scientific (Waltham, MA, USA). The polyvinylidene fluoride (PVDF) membrane was purchased from Bio-Rad (Hercules, CA, USA). Anti-β-actin, anti-cyclin A, and anti-cyclin E antibodies were obtained from Santa Cruz Biotechnology (Dallas, TX, USA). Anti-CD3 and anti-CD28 antibodies were purchased from Bioxcell (West Lebanon, NH, USA). Anti-CD69 and anti-CD25 conjugated with APC were obtained from eBiosciences (San Diego, CA, USA). Anti-caspase 3, anti-caspase 7, anti-caspase 8, anti-caspase 9, anti-TAK1, anti-IKKα, anti-p65, anti-poly (ADP-ribose) polymerase (PARP), anti-IκBα, anti-ERK, anti-p38, anti-JNK, and anti-c-Jun were purchased from Cell Signaling Technology (Danvers, MA, USA). Antibodies against phosphorylated TAK1, IKKα, ERK, p38, JNK, and c-Jun antibodies were also obtained from Cell Signaling Technology.

### 4.6. MTT Assay

Jurkat T cells (1 × 10^4^/well in a 96-well plate) were seeded and treated with the indicated concentration (0–40 μM) of diketoacetonylphenalenone for 24 h. After incubation, cells were incubated with MTT (500 μg/mL) for 1.5 h. Supernatants were removed, formazan crystals were dissolved with 150 μL of dimethyl sulfoxide, and the plate was gently shaken. Each well was read at 540 nm and OD values were obtained to calculate cell viability. Cell viability is presented as a percentage of the control.

### 4.7. Assessment of the Expression of Annexin V and Caspase 3/7 by IncuCyte^®^

Jurkat T cells (1 × 10^4^/well in a 96-well plate) were seeded and treated with the indicated concentration (0–40 μM) of diketoacetonylphenalenone for 24 h. Jurkat T cells were treated with Annexin V and caspase 3/7 before incubation. After incubation, microscopic images for confluency (differential interference contrast), Annexin V (green fluorescence), and caspase 3/7 (red fluorescence) were obtained using an IncuCyte^®^ imaging system (Sartorius, Ann Arbor, MI, USA). Cell confluency and the integrated fluorescence intensity of Annexin V and caspase 3/7 were automatically analyzed using IncuCyte^®^ software and the percentage of the control was calculated for bar graph presentation.

### 4.8. Western Blot Analysis

Jurkat T cells treated with the indicated conditions were harvested and lysed for Western blotting using radioimmunoprecipitation assay (RIPA) buffer (Sigma Chemical Co.). Cells were lysed in RIPA buffer for 20 min on ice and the solution was centrifuged at 14,000 rpm for 20 min at 4 °C. When nuclear extracts were separated from whole lysates, the NE-PER kit was used following the manufacturer’s instructions. Approximately 30 μg of lysate was loaded on 8–12% sodium dodecyl sulfate (SDS)-polyacrylamide gel electrophoresis gels for separation and separated proteins were transferred on PVDF membranes. After transfer, membranes were blocked with 5% skim milk for 1 h and rinsed with Tris-buffered saline containing 0.1% Tween 20 (TBS-T). Membranes were incubated with the indicated primary antibodies in 3% skim milk in TBS-T overnight at 4 °C (dilution factor was 1 to 1000). Excess primary antibodies were discarded with TBS-T and membranes were incubated with 0.1 μg/mL peroxidase-labeled secondary antibodies (against rabbit or mouse) for 2 h. After three washes with TBS-T, bands were visualized using ECL Western blotting detection reagents (Thermo Fisher Scientific) and an ImageQuant LAS 4000 (GE Healthcare, Chicago, IL, USA). All detected bands were normalized to β-actin (whole lysate or cytosolic extracts) and PARP (nuclear extracts). Representative images from three independent experiments are shown in Figure 3 and Figure 7.

### 4.9. T Cell Stimulation

Jurkat T cells were stimulated using two different methods in the present study. For TCR-mediated stimulation, Jurkat T cells were seeded on plates coated with anti-CD3 antibodies (20 μg/mL) overnight at 4 °C and treated with soluble anti-CD28 antibodies (7 μg/mL). For mimicked stimulation of the early signal transduction of T cells, Jurkat T cells were treated with PMA (100 nM) and A23187 (1 μM).

### 4.10. Conventional PCR and Quantitative PCR

To measure the mRNA levels of *il2* in Jurkat T cells, total RNA was isolated using TRIZOL reagent (JBI, Korea) and reverse transcription was performed using RT PreMix. Conventional PCR conditions were as follows: 30 cycles of denaturation at 94 °C for 5 s, annealing at 60 °C for 30 s, and extension at 72 °C for 30 s, followed by denaturation at 72 °C for 7 min. The PCR product was loaded onto 2% SDS gels and bands were detected by UV. For quantitative real-time PCR analysis, the DNA Engine Opticon 1 continuous fluorescence detection system (MJ Research, Waltham, MA, USA) with SYBR Premix Ex Taq (Takara, Japan) was used. The total reaction volume was 10 μL, containing 1 μL of cDNA or control and gene-specific primers. Each PCR reaction was performed using the following conditions: 94 °C for 30 s, 60 °C for 30 s, 72 °C for 30 s, and plate read (detection of fluorescent product) for 40 cycles, followed by 7 min of extension at 72 °C. Melting curve analysis was performed to characterize the dsDNA product by slowly increasing the temperature (0.2 °C/s) from 65 °C to 95 °C with fluorescence data collected at 0.2 °C intervals. The mRNA levels of inflammatory cytokines normalized to *gapdh* were expressed as fold changes. The fold changes in gene expression were calculated using the following equation: fold change = 2 − ΔΔCT, where ΔΔCT = (CT *il2* − CT *gapdh*) at time x − (CT *il2* − CT *gapdh*) at time 0 h. Here, time x represents any time point and time 0 represents the 1 × expression of *il2* in control cells normalized to *gapdh*. Primers used for each gene were as follows (forward and reverse primers, respectively): human *il2*, 5′-CAC GTC TTG CAC TTG TCA C-3′ and 5′-CCT TCT TGG GCA TGT AAA ACT-3′; human *gapdh*, 5′-CGG AGT CAA CGG ATT TGG TCG TAT-3′ and 5′-AGC CTT CTC CAT GGT GGT GAA GAC-3′.

### 4.11. ELISA

To determine human IL-2 production from activated T cells, IL-2 levels were measured, using a human IL-2 ELISA kit following the manufacturer’s instructions, from supernatants collected from activated T cells under different conditions.

### 4.12. Proliferation Assay

Jurkat T cells (1 × 10^5^/well in a 24-well plate), pre-stained with 1 μM CFSE for 30 min, were pretreated with the indicated concentration of diketoacetonylphenalenone for 1 h. The cells were then stimulated with anti-CD3/CD28 antibodies for 12 h and 24 h. Resting Jurkat T cells were acquired as the positive control at 0 h. After stimulation, cells were harvested, CFSE fluorescence was measured by flow cytometry, and histograms were created. The MFIs of CFSE and CFSE-positive cells were analyzed using the histograms and are presented as bar graphs.

### 4.13. Determination of Surface Molecule Expression on T cells

Jurkat T cells (1 × 10^5^/well in a 24-well plate) pretreated with the indicated concentration of diketoacetonylphenalenone for 1 h were stimulated with anti-CD3/CD28 antibodies. After incubation, cells were stained with anti-CD69 or anti-CD25 antibodies conjugated with APC for 30 min at 4 °C. Cells were acquired by flow cytometry and representative histograms are shown. The MFIs of CD69 and CD25 were analyzed using histograms and are presented as bar graphs.

### 4.14. Statistics

One-way ANOVA was used to determine significance (*p* value). Mean values ± SEM were calculated from the acquired data of three independent experiments performed on separate days and are presented in graphs with *p* values (*). * represents significant differences compared with mock-treated controls at *p* < 0.05.

## Supplementary Materials

The following are available online, Figure S1: ^1^H-NMR spectrum of diketoacetonylphenalenone (DAP) (CD_3_OD, 850 MHz); Figure S2: ^13^C NMR spectrum of DAP (CD_3_OD, 212.5 MHz); Figure S3: UV spectrum of DAP; Figure S4: UV chromatogram of LC/MS (detection wavelength was set as 254 nm) of DAP; Figure S5: (A) UV chromatogram of LC/MS (detection wavelength was set as 254 nm) of mycelia and liquid broth of *P. herquei* FT729. (B) UV chromatogram of LC/MS (detection wavelength was set as 254 nm) of EtOAc and *n*-BuOH fractions.
