# Supplementary material for: Diketoacetonylphenalenone, Derived from Hawaiian Volcanic Soil-Associated Fungus Penicillium herquei FT729, Regulates T Cell Activation via Nuclear Factor-κB and Mitogen-Activated Protein Kinase Pathway
Source: Molecules. 2020 Nov 17;25(22):5374. doi: 10.3390/molecules25225374 (PMC7698495; doi:10.3390/molecules25225374)
Supplement: Supplementary file 1 [file molecules-25-05374-s001.pdf]

Supplementary Material

Diketoacetonylphenalenone, derived from Hawaiian volcanic soil-associated fungus *Penicillium herquei* FT729, regulates T cell activation via nuclear factor-κB and mitogen-activated protein kinase pathway

Hyun-Su Lee [1], Jae Sik Yu [2], Ki Hyun Kim [2,*] and Gil-Saeng Jeong [1,*]

[1] College of Pharmacy, Keimyung University, Daegu 42601, Korea; hyunsu.lee@kmu.ac.kr
[2] School of Pharmacy, Sungkyunkwan University, Suwon 16419, Korea; jsyu@bu.edu

* Correspondence: khkim83@skku.edu (K.H.K.); Tel.: +82-(31)-290-7700 (K.H.K.), gsjeong@kmu.ac.kr (G.S.J.); Tel.: +82-(53)-580-6649 (G.S.J.)

Figure S1 : ^{1}H-NMR spectrum of diketoacetonylphenalenone (DAP) (CD_3OD, 850 MHz)

Figure S2 : ^{13}C NMR spectrum of DAP (CD_3OD, 212.5 MHz)

Figure S3 : UV spectrum of DAP

Figure S4 : UV chromatogram of LC/MS (detection wavelength was set as 254 nm) of DAP

Figure S5 : (A) UV chromatogram of LC/MS (detection wavelength was set as 254 nm) of mycelia and liquid broth of *P. herquei* FT729. (B) UV chromatogram of LC/MS (detection wavelength was set as 254 nm) of EtOAc and *n*-BuOH fractions.

Figure S1. The ^{1}H NMR spectrum of DAP (CD_3OD, 850 MHz)

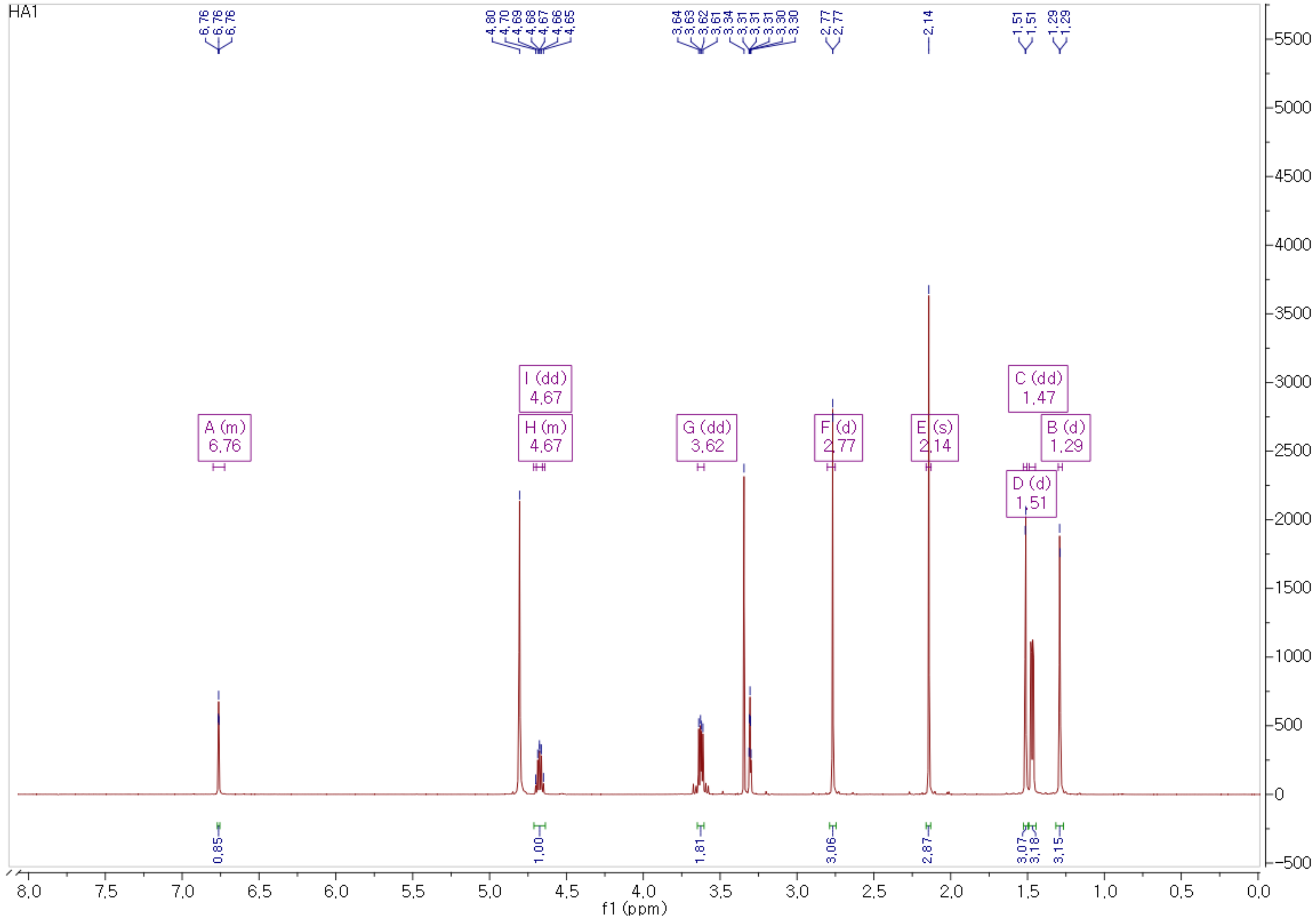

Figure S2. The ^{13}C NMR spectrum of DAP (CD_3OD, 212.5 MHz)

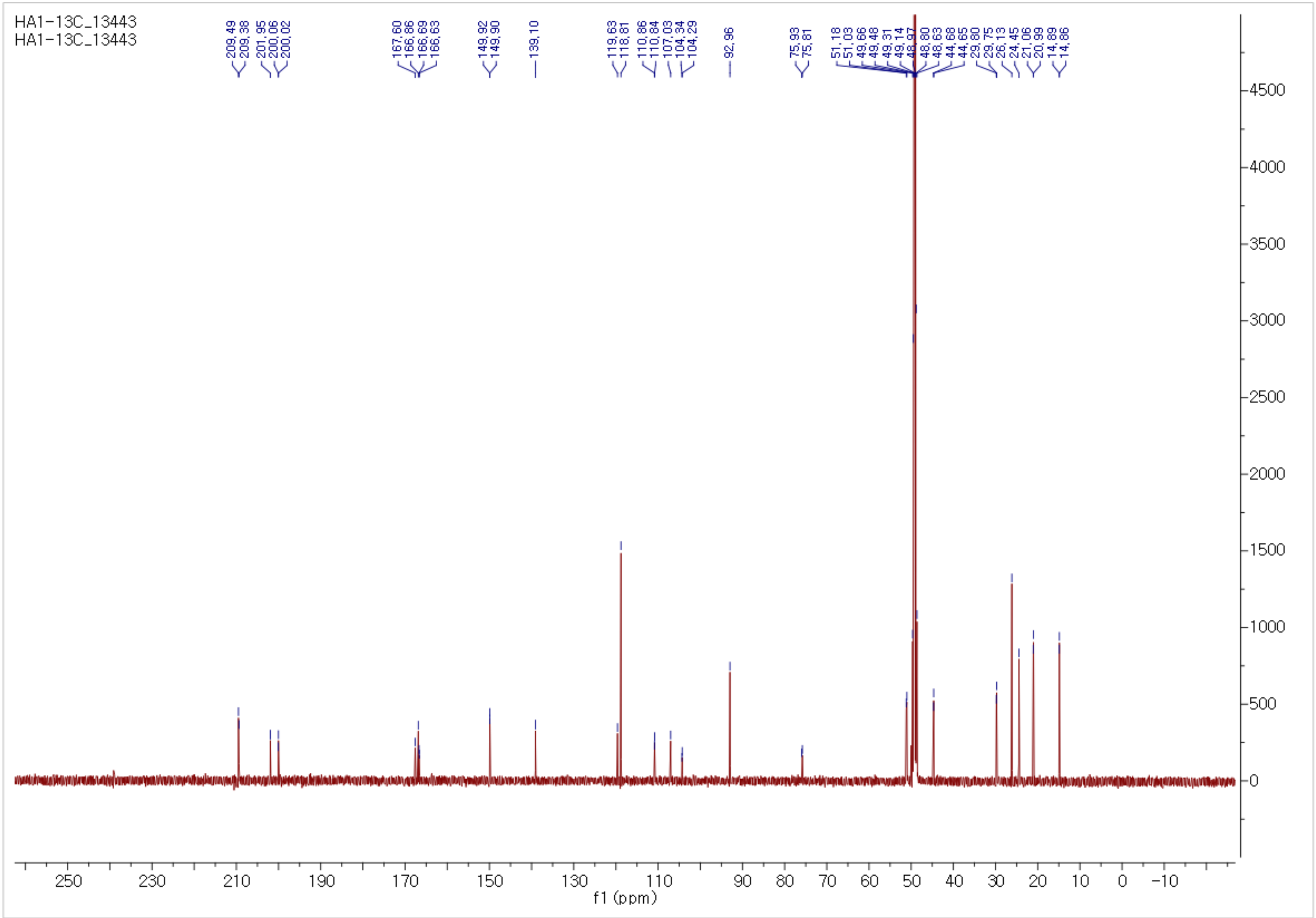

Figure S3. UV spectrum of DAP

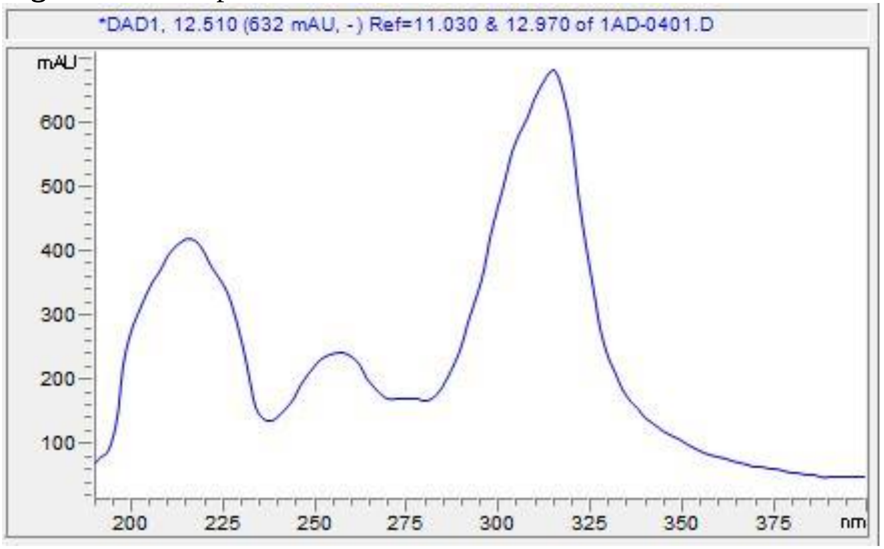

Figure S4. UV chromatogram of LC/MS (detection wavelength was set as 254 nm) of DAP

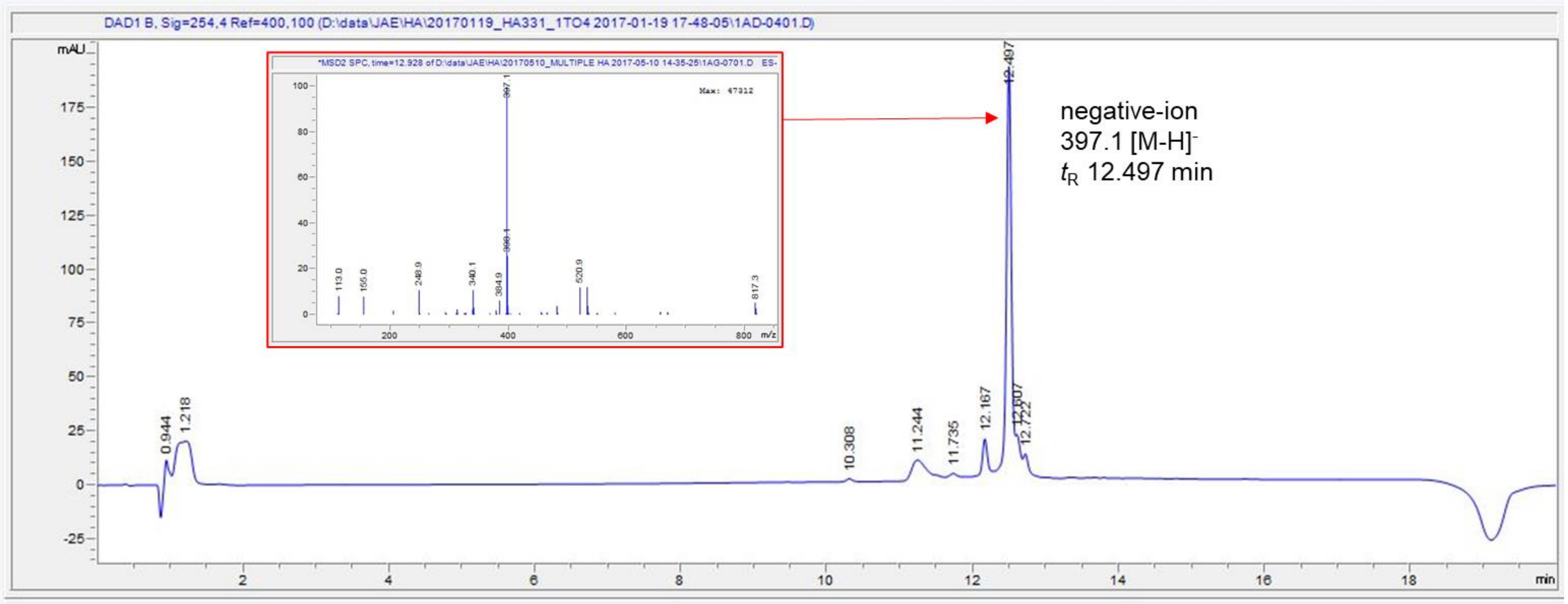

LC/MS analysis

LC/MS analysis for DAP was performed by LC/MS (Agilent Technologies, Santa Clara, CA, USA) using a LC-MS Agilent 1200 Series analytical system equipped with a photodiode array (PDA) detector combined with a 6130 Series ESI mass spectrometer. Analysis was performed by injection of 10 μL of DAP using a Kinetex C18 column (2.1 × 100 mm, 5 μm; Phenomenex, Torrance, CA, USA) set at 25 ℃. The mobile phase consisting of formic acid in H_2O [0.1% (v/v)] (A) and methanol (B) was delivered at a flow rate of 0.3 mL/min by applying the following programmed gradient elution: 10%-100% (B) for 10 min, 100% (B) for 1 min, 100% (B) isocratic for 5 min, and then 0% (B) isocratic for 5 min, to perform post-run reconditioning of the column.

Figure S5. (A) UV chromatogram of LC/MS (detection wavelength was set as 254 nm) of mycelia and liquid broth of *P. herquei* FT729. (B) UV chromatogram of LC/MS (detection wavelength was set as 254 nm) of EtOAc and *n*-BuOH fractions.

(A)

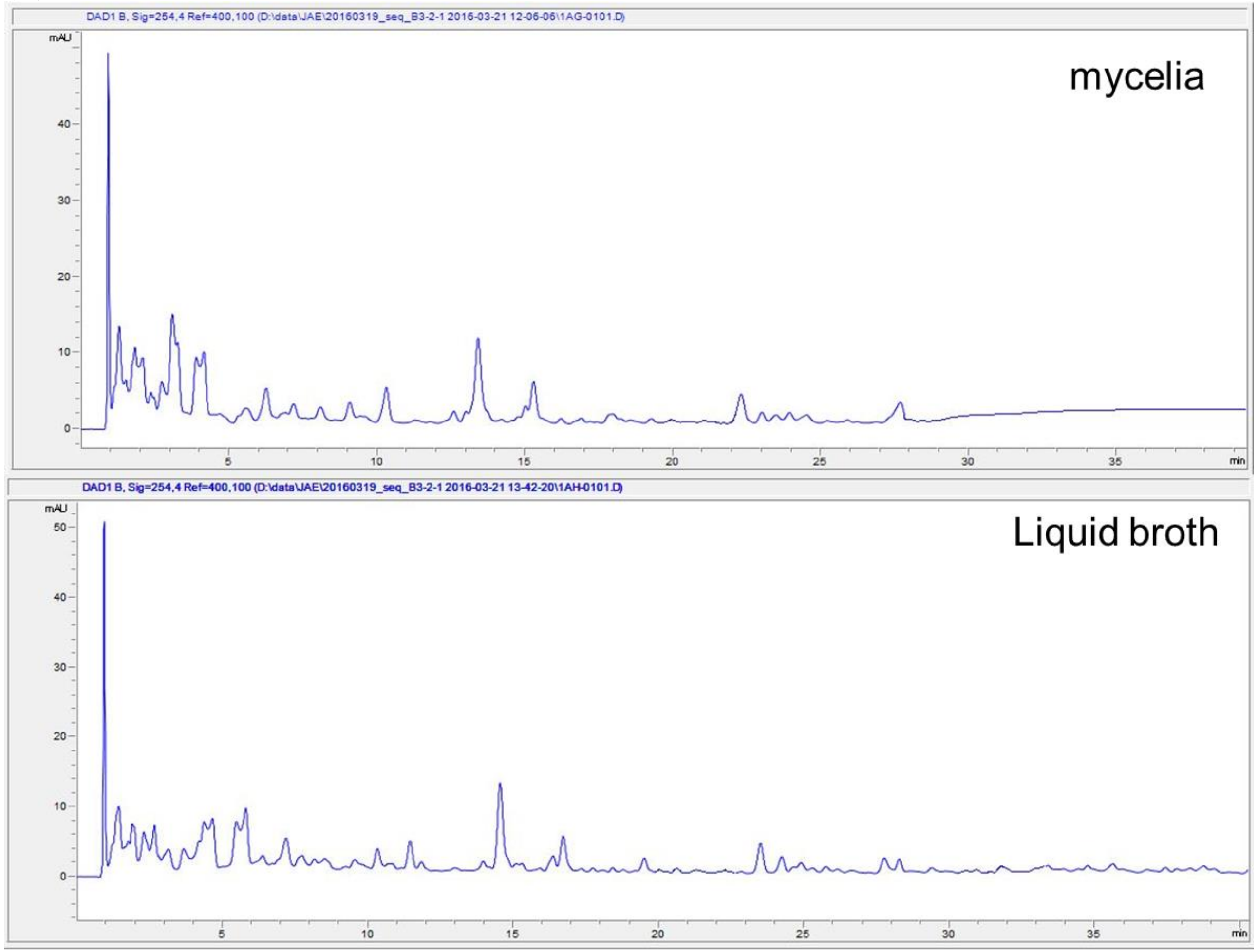

(B)

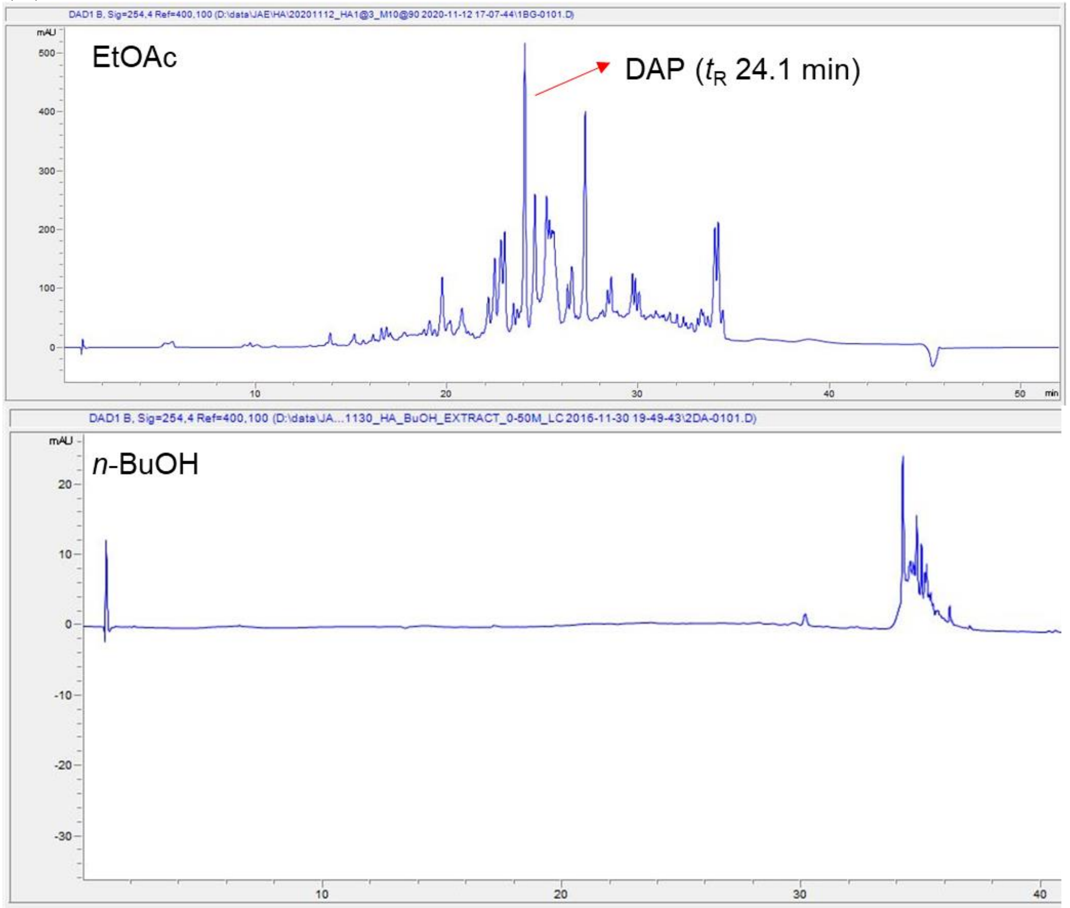

LC/MS analysis

LC/MS analysis for mycelia or liquid broth of *P. herquei* FT729 was performed by the same LC/MS equipment (Agilent Technologies, Santa Clara, CA, USA). Analysis was performed by injection of 10 μL of samples using a Kinetex C18 column (2.1 × 100 mm, 5 μm; Phenomenex, Torrance, CA, USA) set at 25 ℃. The mobile phase consisting of formic acid in H_2O [0.1% (v/v)] (A) and methanol (B) was delivered at a flow rate of 0.3 mL/min by applying the following programmed gradient elution: 0%-100% (B) for 30 min, 100% (B) for 1 min, 100% (B) isocratic for 10 min, and then 0% (B) isocratic for 10 min, to perform post-run reconditioning of the column.
